# Re-evaluating the evidence for a universal genetic boundary among microbial species

**DOI:** 10.1038/s41467-021-24128-2

**Published:** 2021-07-07

**Authors:** Connor S. Murray, Yingnan Gao, Martin Wu

**Affiliations:** Department of Biology, University of Virginia, Charlottesville, VA USA

**Keywords:** Classification and taxonomy, Speciation, Bacterial evolution

Arising from Jain et al. *Nature Communications* 10.1038/s41467-018-07641-9 (2018)

A fundamental question in studying microbial diversity is whether there is a species boundary and if the boundary can be delineated by a universal genetic discontinuity. To address this question, Jain et al. computed the pairwise average nucleotide identity (ANI) of 91,761 microbial (bacterial and archaeal) genomes (the 90K genome dataset) and found that the ANI values from the 8 billion comparisons follow a strong bimodal distribution with a wide gap between 83 and 95%^1^. As a result, the authors concluded that a clear genetic discontinuum and species boundary were evident from the unprecedented large-scale ANI analysis, and claimed that “it (the 95% ANI threshold) represents an accurate threshold for demarcating almost all currently named prokaryotic species”. We argue that the paper’s conclusion of a universal genetic boundary among named species in the current NCBI taxonomy is questionable and resulted from the substantial biased sampling in genome sequencing, and caution against being overly confident in using 95% ANI for microbial species delineation as the high benchmarks reported in the paper were inflated by using highly redundant genomes.

To demonstrate our point, we first show that biased within-species sampling can generate the bimodal distribution of ANI observed in the original paper even when the speciation rate is constant and the genetic diversity is continuous. We simulated continuous genetic diversity using a 3000-tip phylogenetic tree that diversifies at a constant rate, which we estimated from a genome tree of 3000 bacterial genomes that represents the phylogenetic diversity in the 10,616 NCBI RefSeq complete bacterial genomes (the 10K genome dataset). We then calculated ANI between tips using a function that accurately captures the relationship between ANI and the branch length in the real data (Supplementary Fig. 1). Because the diversification rate is constant, the branch lengths follow an exponential distribution expected from a Poisson process. As expected, the frequency of ANI declined monotonically when ANI increased (Fig. 1a). However, when only 30 tips (1% of the tips) were sampled with a protocol that emulated within-species sampling bias (each tip has two very closely related genomes sequenced), the ANI distribution became bimodal (Fig. 1b). Although our simulation using a simplistic model does not disprove the existence of a universal genetic boundary, it demonstrates the possibility that limited within-species sampling bias alone can create the bimodal distribution when genetic diversity is continuous.

**Fig. 1:**
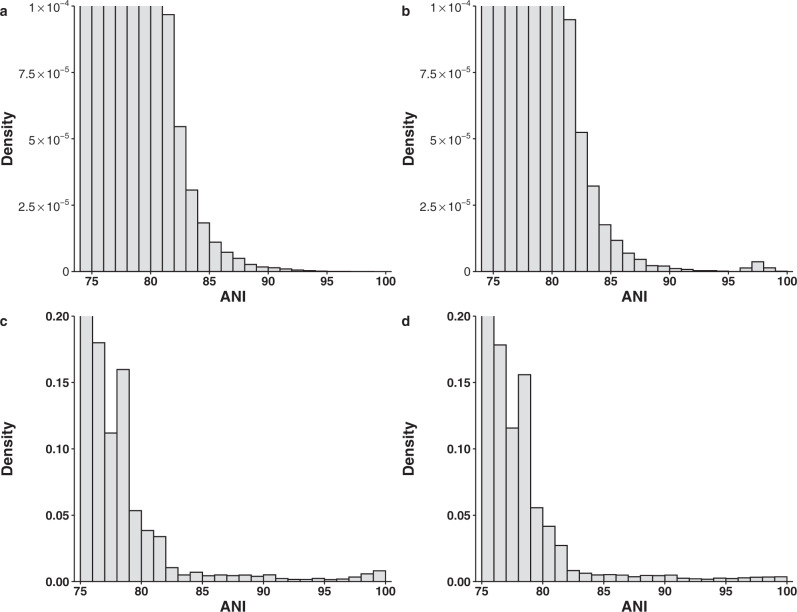
Distribution of ANI values. Top panel: from the phylogenetic simulations. **a** Comparisons between 3000 taxa simulated using a phylogenetic tree with 3000 tips and a constant rate of diversification. **b** Comparisons in the same dataset except that 30 of 3000 taxa each have two very closely related genomes sequenced. Bottom panel: from genomes subsampled from the 90K genome dataset. **c** Two genomes were randomly selected from each of the 397 named species with ≥10 genomes. **d** Two phylogenetic representative genomes were selected for each of the 397 named species with ≥10 genomes.

In the original 90K genome dataset, 33% of named species have been sequenced at least twice. As cultivation bias is widespread and strains of medical and economic interest are heavily favored in our genome sequencing efforts, next we show that there is substantial within-species sampling bias in the genome datasets. For the model organism *Escherichia coli*, its 602 complete genomes in the 10K genome dataset only represent 22% of the diversity captured by the 16S rRNA gene in the GreenGene database (Supplementary Fig. 2). For species with at least 100 genomes, on average the first 75.8% (range: 50.9–97.7%) of dropped genomes contribute <5% of the genetic diversity of the species as measured by the branch length (Supplementary Fig. 3). As demonstrated in our phylogenetic simulation, these highly redundant genomes can create the bimodal distribution even when genetic diversity is continuous. Contrary to the authors’ claim and from a purely statistical point of view, randomly subsampling (e.g., sampling five genomes from the same species as done in the original study^1^) from a biased dataset will not correct for the pre-existing sampling bias. When two genomes were sampled randomly from each of the 397 named species with ≥10 genomes in the 90K dataset, the bimodal distribution was evident (Fig. 1c). However, when we reduced the within-species sampling bias by selecting two phylogenetically representative genomes from the same dataset, the distribution flattened near the end (Fig. 1d), further demonstrating that the bimodal distribution can be caused by the widespread sampling bias within species. In fact, the within-species sampling bias has increased over time when more strains were sequenced based on their medical and economic relevance and not on their phylogenetic positions (Supplementary Fig. 4), thereby producing the consistent bimodal distributions of ANI over different periods of time as observed in the original study^1^.

The authors indicated that only 0.2% of the 8 billion ANI values are between 83 and 95% and suggested that such a wide gap is evidence of a clear genetic boundary in microbial genomes. In our phylogenetic simulation of 3000 genomes with continuous genetic diversity, only 0.11% of ANI values span the same region. This is expected because without biased sampling, the vast majority of all pairwise comparisons for a genome will be with distantly related genomes, whose number will always be much larger than the number of closely related taxa. As a result, the fraction of ANI values in the intermediate range [83–95%] will always be marginal for a decently sized tree (>50 tips) and will decrease when the number of genomes increases (Supplementary Fig. 5). Our result shows that the low density of ANI values in the gap region does not necessarily indicate a genetic boundary. Instead, it could simply reflect the hierarchical structure of the phylogenetic relationship. Consistent with our finding, a recent analysis of ~150,000 bacterial and archaeal genomes shows the interspecies ANIs between closest representatives within a genus are nearly evenly distributed between 78 and 95% ANI and there is no genetic discontinuum in this region^2^.

Previous studies have advocated using an ANI cutoff to demarcate bacterial species^3–6^. Based on the unprecedented large-scale ANI analysis of the study, Jain et al. claimed that using the 95% ANI criterion led to both high recall and precision rates (>98.5%) in demarcating named prokaryotic species in the current NCBI taxonomy^1^. However, both benchmarks were calibrated with ~78k named genomes that were highly redundant. For example, the dataset contained more than 5000 *E. coli* genomes. As such, the recall and precision rates can be misled by the overly sampled genomes. To better assess the performance of using 95% ANI for species demarcation, we queried the 3000 representative genomes against the 10K dataset. We found the recall and precision rates were much lower, at 73.4% and 83.3% respectively. The large impact of the extreme sampling bias on the benchmarks is best illustrated by the authors’ own finding that excluding the *E. coli* vs. *Shigella spp*. comparison alone substantially increased their overall precision from 93.1 to 98.7%. Plotting of the intraspecific ANI for intensely sequenced species in the 90K dataset (61 named species with ≥100 genomes, representing 6 phyla) shows that a universal boundary at 95% ANI clearly does not exist in these species, as ANI values drop well below 95% with few exceptions (Fig. 2). The high density above the 95% threshold could be an artifact caused by comparing highly redundant genomes within the same species.

**Fig. 2:**
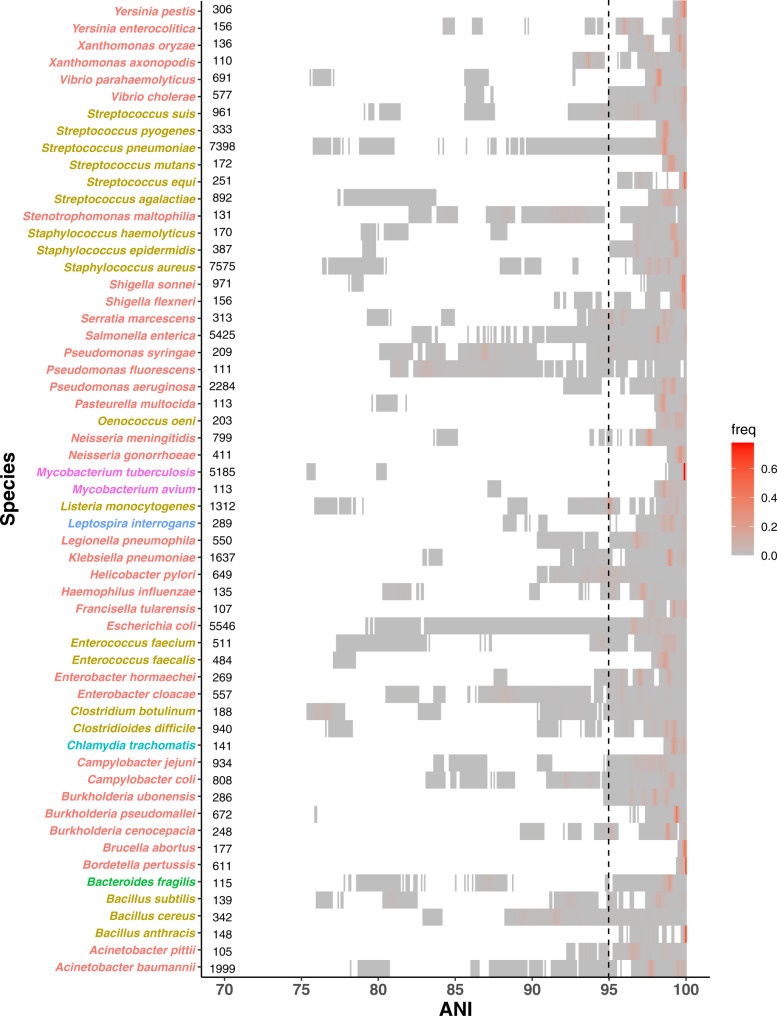
The distribution of ANI for 61 named species with ≥100 genomes in the 90K genome dataset. Each block represents a bin of ANI values between pairs of genomes within the same species and is colored by the ANI density in the bin. Species are colored by phylum. The numbers next to the species names are the numbers of genomes in the 90K dataset for the species. The vertical dotted line represents the 95% ANI threshold.

Although having no cultivation bias, metagenome assemblies do favor abundant strains over rare ones. This bias can lead to the appearance of genetic clusters when diverse but rare strains in the community are excluded from the assembled genomes. In addition, a rare strain in one environment can be abundant in another. Furthermore, even if a genetic cluster does exist in one environment, it does not necessarily delineate the genetic boundary of the species in general. For example, various *E. coli* strains spanning a continuum of diversity live in the gut, water, and soil^7^. Analyzing gut metagenomic data alone will most likely reveal a tight genetic cluster of *E. coli*. However, this genetic cluster does not represent the genetic boundary of *E. coli* as a species because it excludes the environmental *E. coli* strains. It can be argued that *E. coli* is an exception, but it is also well known that many bacterial species are “generalists” that live in a wide range of habitats. One potential solution is to narrow down our species definition to accommodate the local genetic clusters, but doing so will require substantially overhauling the current taxonomy and change the subject of this debate, the named species in the current taxonomy. Unless low abundance strains are readily recovered (e.g., through read recruitment to a reference genome) and metagenomic sequences from different types of environments are compared, there are also potential pitfalls associated with demonstrating the genetic boundary of currently named species using metagenome-assembled genomes^8^. Interestingly, several metagenomic studies have revealed genetic continuum in nature^9–11^.

There is much evidence against the existence of a universal genetic boundary for microbial species. First, the molecular substitution rate is highly variable across species. Secondly, selection and recombination are thought to be the main cohesive forces driving the formation of genetic clusters. Although recombination rate can be influenced by sequence similarity, there is no correlation between the recombination rate and ANI in bacteria^12^, as recombination can also be affected by physical and ecological barriers. Microbes living in narrow ecological niches and with limited dispersal rate (e.g., obligate intracellular bacteria) may develop genetic clusters. On the other hand, free living microbes exploring different habitats and mixing by dispersal are more likely to exhibit a genetic continuum^13^. Selection is unlikely to produce a universal genetic boundary either, as microbial species are unique in nature, with each species subject to its own evolutionary and ecological forces^14^.

In summary, our study shows that the genetic boundary perceived in the original paper can be explained by persistent within-species sampling bias from historic and current genome sequencing efforts. A more balanced analysis of the present genomic data shows that although genetic clusters may exist in individual species, we find no evidence of a universal genetic boundary among named microbial species in the most recent NCBI taxonomy.

## Methods

### Genome datasets

Two genome datasets were used in this study. The first is the 90K genome dataset from the original paper^1^. It contains both complete and draft bacterial and archaeal genomes. The second dataset consists of 10,616 complete bacterial genomes downloaded from the NCBI RefSeq database on September 6, 2018 (10K genome dataset). From each genome in the 10K dataset, we identified 31 universal protein-coding marker genes using AMPHORA2^15^ and constructed a bacterial genome tree based on the concatenated and trimmed protein sequence alignment of the marker genes using FastTree^16^. Treemmer (version 0.3)^17^ was used to choose 3000 representative genomes that maximized the phylogenetic diversity in the 10K genome dataset.

### Average nucleotide identity (ANI)

The ANI values for the 90K genome dataset were downloaded from the original study. For the 10K genome dataset, the 3000 representative genomes were compared against the full 10K dataset using FastANI (version 1.2).

### Modeling the relationship between branch length and ANI

The 3000 representative genomes were used to model the empirical relationship between ANI and branch length. The median of ANI was calculated across binned branch lengths (bin width: 0.05 substitution/site) to use as the actual data to fit the relationship between ANI and branch length *l* through the function $ANI( \% )=k+\frac{\alpha \cdot (100-k)}{{l}^{s}+\alpha }$, where *k*, *s* and *α* are shape parameters to be estimated. Minimization of the sum of squares error was performed using the *optim* function in R. The best fit parameters for our data are *α* = 0.075, *k* = 73.94, and *s* = 0.63. Branch lengths >2.5 substitutions/site were removed because of the lack of data points.

### Simulation of continuous genetic diversity and biased within-species sampling

The *rtree* function in the *ape* package in R was used to simulate a random phylogenetic tree of 3000 tips, with its branch lengths following an exponential distribution with a constant rate of 19.2, estimated from the genome tree of the 3000 representative genomes. Using the formula described above, the ANI value between a pair of genomes was computed from the branch length between them. To simulate biased sampling within species, a random tip was chosen and two descendants were added to that tip, with the branch length from the tip to the descendant sampled from the same exponential distribution, but its value restricted to the bottom 1% of the distribution. This procedure was repeated on the remaining 2999 tips until *n* tips were processed. Each simulation was run with ten replicates.

### Assessing the within-species sampling bias

Species with ≥10 genomes were selected from the 10K genome dataset. For each species, a subtree compiling the respective genomes was extracted from the full phylogeny of 10,616 genomes and Treemmer was used to iteratively remove one tip of the tip-pair with the shortest branch length until three tips remained. The remaining total branch length of the tree was divided by the total branch length of the initial tree to calculate the relative tree length at each iteration. *Rickettsia japonica* and *Chlamydia muridarum* were removed from this analyses because their genomes have identical marker sequences and branch lengths equal to zero.

To further evaluate the within-species sampling bias, we tested how much known genetic diversity is recovered by the complete genomes, using *E. coli* as an example. We extracted 868 unique 16S rRNA gene sequences with no ambiguous bases from 602 complete *E. coli* genomes in the 10K genome dataset and BLAST searched them against 8655 *E. coli* 16S rRNA gene sequences from the GreenGene 13.8 database. A match was defined as a pair of sequences with 100% identity for their entire sequences. The matched GreenGene 16S rRNA sequences were then mapped to the 99% OTUs (operational taxonomic units) of the GreenGene database. The total branch length covered by the mapped OTUs in the 16S rRNA tree of 44 *E. coli* 99% OTUs was calculated to estimate the coverage of *E. coli* diversity by complete genomes.

### Subsampling of two genomes

For named species with ≥10 genomes in the 90K dataset, two genomes were sampled from each species either randomly or by selecting the pair with the lowest ANI value. Among all pairwise comparisons within the species, the pair with the lowest ANI best represents the phylogenetic diversity of the species.

### Benchmark the performance using 95% ANI for species demarcation

Using the 3000 representative genomes as the query, we ran FastANI against the full 10K genome dataset. For each query genome, the subject genome with the maximum ANI value was used to benchmark the performance of using the 95% ANI threshold to demarcate bacterial species. A true positive is a query-subject pair belonging to the same species and having an ANI ≥ 95%. A false positive is a genome pair of different species with an ANI ≥ 95%, and a false negative is a genome pair of the same species with an ANI < 95%. Precision was calculated by: the number of true positive/(number of true positive + number of false positive) and recall was calculated by: the number of true positive/(number of true positive + number of false negative).

### Reporting summary

Further information on research design is available in the Nature Research Reporting Summary linked to this article.

## Supplementary information

Supplementary Information

Reporting Summary

## Data Availability

Genome sequences were downloaded from the NCBI RefSeq Database (https://ftp.ncbi.nlm.nih.gov/genomes/refseq). FastANI values were downloaded from the server listed in the original paper. All the other data are available for download at https://github.com/wu-lab-uva/FastANI-Rebuttal.

## Code availability

All R code is available for download at https://github.com/wu-lab-uva/FastANI-Rebuttal.
