# Supplementary material for: Re-evaluating the evidence for a universal genetic boundary among microbial species
Source: Nat Commun. 2021 Jul 7;12:4059. doi: 10.1038/s41467-021-24128-2 (PMC8263626; doi:10.1038/s41467-021-24128-2)
Supplement: Supplementary file 1 — Supplementary Information [file 41467_2021_24128_MOESM1_ESM.pdf]

Supplementary Information for:

Re-evaluating the evidence for a universal genetic boundary among microbial species

Murray et al.

Supplementary Figures 1-5

Supplementary Figures

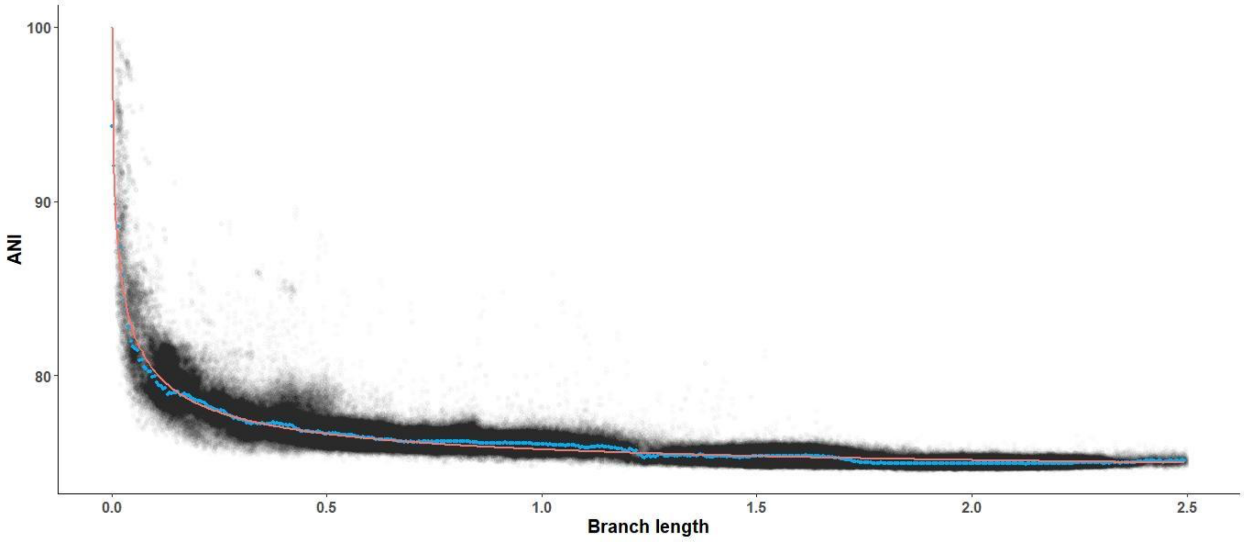

Supplementary Figure 1. The relationship between the branch length and ANI. Shaded black points are pairwise comparisons between the 3,000 representative genomes. Blue points are the median ANI for each branch length bin (bin width: 0.05 substitution/site). Orange line is the fitted curve. Branch lengths greater than 2.5 were removed because of the lack of data points.

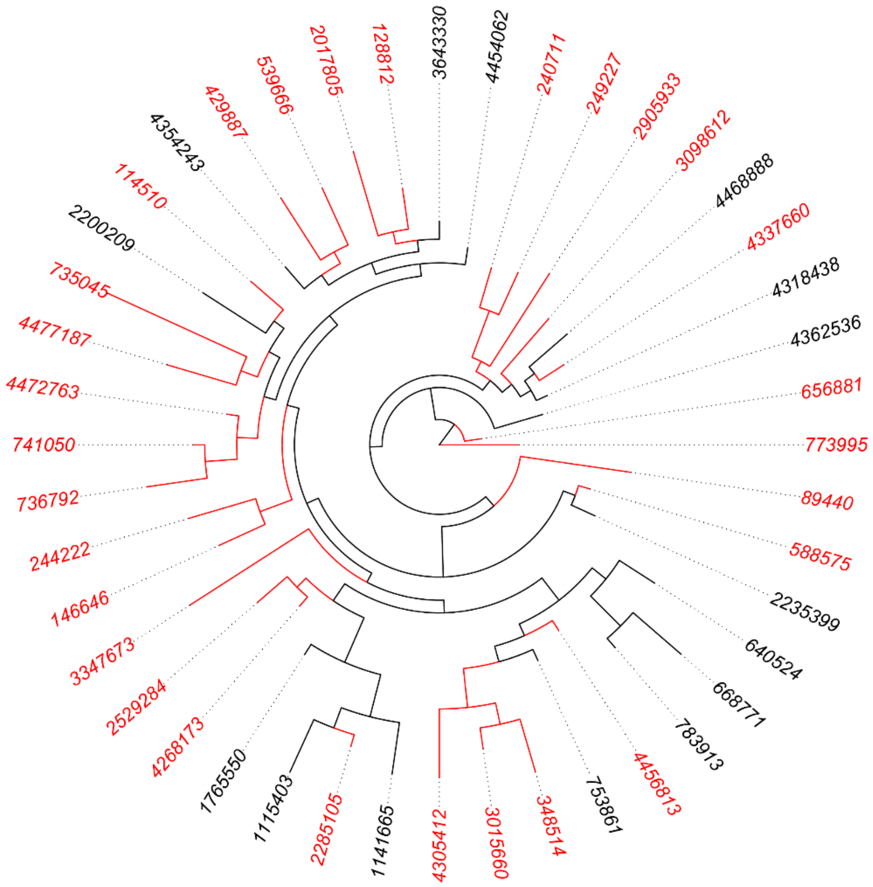

Supplementary Figure 2. Incomplete and biased coverage of *E. coli* diversity by complete genomes. Of 44 *E. coli* 99% OTUs in the phylogeny of the 16S rRNA gene, only 15 (in black) are represented by the 602 complete *E. coli* genomes in the 10K genome dataset, and only 22% of the total branch length in the phylogeny is covered by these 15 OTUs. The branch length was square-root transformed to better show the short branches in the figure.

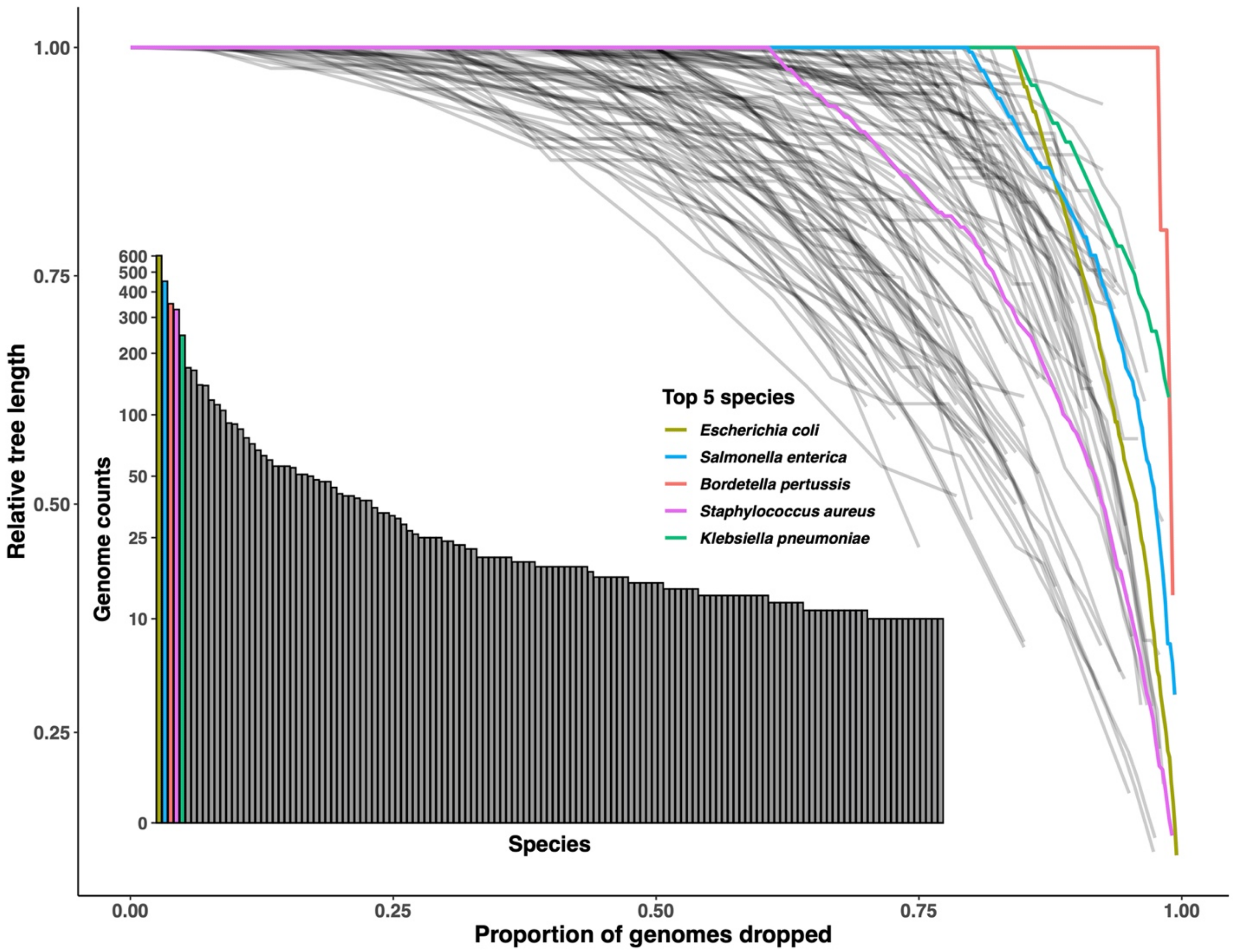

Supplementary Figure 3. Widespread within-species sampling bias revealed by the Treemmer analysis. Treemmer iteratively drops genomes contributing the least amount of diversity within a phylogenetic tree until only three genomes remain. For each species, the relative tree length (RTL) is plotted against the proportion of genomes dropped at each iteration. Colored lines are the 5 most sequenced species in the 10K genome dataset. Shaded black lines are species with ≥10 genomes. The bar chart shows species ranked by the number of genomes sequenced.

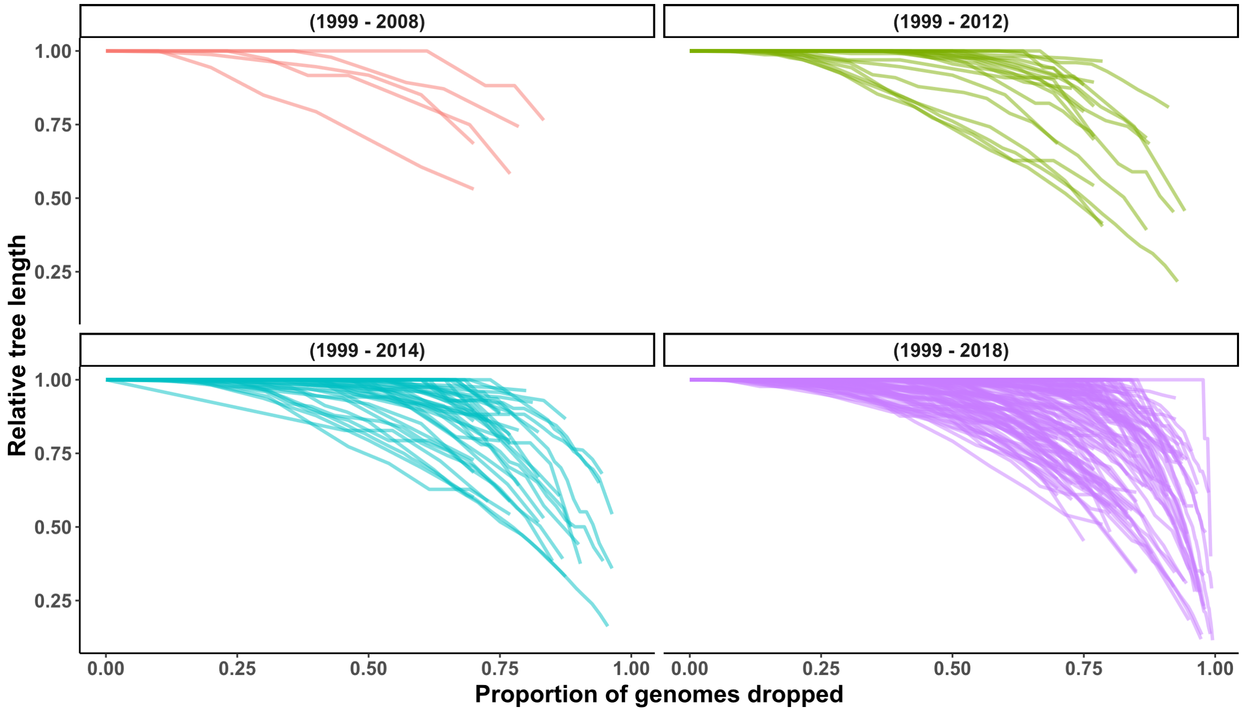

Supplementary Figure 4. Relative tree length (RTL) decay for species with ≥ 10 sequenced genomes in the NCBI RefSeq database in four different time periods.

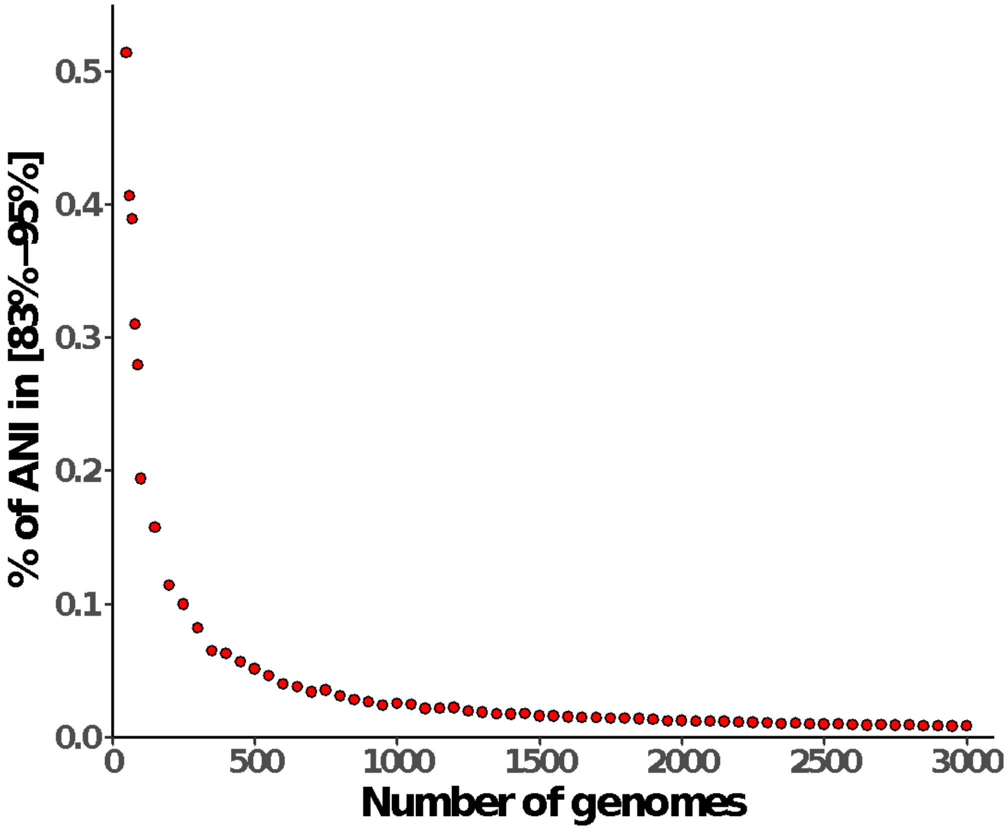

Supplementary Figure 5. The percentage of ANI values in the [83%-95%] range is marginal and has a negative relationship with the number of genomes. A random phylogeny with an exponential distribution of branch lengths was used to simulate continuous genetic diversity across genomes. The pairwise ANI values were calculated from the phylogenetic distances between genomes (see materials and methods). The simulations were run with phylogenies of different sizes ranging from 50 to 3,000 tips.
